# TP53 mutation predicts the poor prognosis of non-Hodgkin lymphomas: Evidence from a meta-analysis

**DOI:** 10.1371/journal.pone.0174809

**Published:** 2017-04-03

**Authors:** Peipei Xu, Xu Liu, Jian Ouyang, Bing Chen

**Affiliations:** Department of Hematology, Nanjing Drum Tower Hospital Clinical College of Nanjing Medical University, Nanjing, Jiangsu Province, People’s Republic of China; Shanghai Jiao Tong University School of Medicine, CHINA

## Abstract

Non-Hodgkin lymphoma (NHL) is a group of malignant hematologic disorders with high heterogeneity. The diagnosis, clinical manifestations, classification, and prognosis of this condition differ among numerous NHL subgroups. The prognostic significance of the mutation of TP53, a tumor suppressor gene involved in cell cycle regulation, should be confirmed in NHL. In this study, our searching strategy and inclusion criteria were implemented, and the pooled hazard ratios (HRs) of the included studies were calculated directly or indirectly. A total of 1,851 patients were enrolled in 22 studies. A meta-analysis was then performed using STATA version 12.0 to confirm the correlation between the status of TP53 mutation and the survival time of patients with NHL. Statistical heterogeneity was assessed with a chi-square-based Q statistical test and Inconsistency index (*I*^2^) statistic. Sensitivity analysis and publication bias were also evaluated. A total of 22 studies were included in our meta-analysis. The pooled HR of the overall survival from 20 studies was 2.30 (95% CI: 1.92–2.76, p = 0.001) with heterogeneity (I^2^ 30.2% p = 0.099). The pooled HR of the progression free survival provided in 5 articles was 2.28 (95% CI: 1.78–2.93, p = 0.001) with heterogeneity (I^2^ 39.8% p = 0.156). No publication bias was found among the included studies, and sensitivity analysis suggested that the combined HRs were stable after any of the studies was excluded from our meta-analysis. This study identified the prognostic significance of TP53 mutation that varied in different NHL subgroups. The group with a mutated TP53 was significantly associated with poor prognosis in patients with NHL. This parameter is a valuable basis for accurate individual therapeutic regimens.

## Introduction

Lymphoma has been commonly detected in developed countries [1], and this condition ranks sixth among cancer diseases affecting males and females in the USA [2]. Although the improvement of the diagnosis, classification, and treatment for NHL results in a decreased death rate, non-Hodgkin lymphoma (NHL) remains the ninth leading cause of cancer-related deaths in the USA [2]. With the heterogeneity and epidemiology of NHL, prognostic factors are necessary to achieve accurate classification and individual treatment regimens.

International prognostic index (IPI) is a widely used clinical prognostic indicator for aggressive NHL [3] and is based on patients treated with combination chemotherapy; however, NHL has been widely treated with a regimen comprising rituximab, cyclophosphamide, doxorubicin, vincristine, and prednisone (R-CHOP). Therefore, the accuracy of IPI may require improvement. Adjusted IPI has been extensively investigated, but an adjusted standard has yet to be established.

Molecular markers have been applied to clinical studies on the evaluation of survival time, pesticide effect, and individualized treatment. For instance, Heselmeyer-Haddad [4] indicated that the amplification of TERC is a necessary condition in cervical cancer development, and its detection can help diagnose low-grade lesions with high progression risks. CYP2C19 mutation is related to a weak response to clopidogre, and Food and Drug Administration (FDA) recommends the detection of CYP2C19 gene type in patients before they are treated with clopidogre [5]. With the deletion of TP53, the short survival time of patients with plasma cell myeloma can be predicted [6]. However, relevant prognostic indicators based on molecular markers for NHL remain insufficient.

An important tumor suppressor gene localized in chromosome 17P13.1, TP53 is considered as the gatekeeper for cellular growth and division [7]. TP53 tumor suppressor is involved in cell cycle processes, including DNA repair, apoptosis, senescence, and autophagy under cellular stress [8, 9]. TP53 mutation significantly affects cancer development because of its function, that is, TP53 regulates cell cycle and transcription to prevent excessive cellular activation.

The significance of TP53 mutation for NHL prognosis is inconsistent in several studies [10] [11]. TP53 mutation is a secondary genetic event in different types of B-cell and T-cell lymphomas [11]. The sample quantity in some reports is also insufficient to confirm the importance of TP53 mutation in NHL prognosis. Thus, further studies should be performed to reveal the relationship between the survival time of patients with NHL and the status of TP53 mutation.

In this research, a meta-analysis was performed to estimate the correlation of TP53 mutation and the survival time of patients with various NHLs and to confirm the value of TP53 mutation as a prognostic indicator for NHL.

## Methods

### Literature search

To ensure retrieval of all relevant studies, we used a broad search strategy with several key words in two electronic databases, including “PubMed” and Web of Science”. The following items were searched: “p53,”“prognosis,” and”lymphoma.” An upper date limit of February 2016 was applied, and no earlier data limitation. After the titles and abstracts were examined to include the studies that may meet the criteria, the full-text reading of the included articles was performed. The references of the included studies were also assessed to obtain additional experimental data.

### Inclusion and exclusion criteria

The inclusion and exclusion criteria were strictly implemented, and the studies meeting the following requirements were included in this document. (1) The study investigated the relationship between TP53 mutation in tumor samples and the overall survival (OS) and progression free survival (PFS) of the NHL. (2) The study provided adequate data to calculate the hazard ratio (HR) of the OS and the PFS along with the corresponding 95% confidence interval (CI). (3) The study used reliable gene mutation technology such as DNA sequencing, polymerase chain reaction-single stranded conformation polymorphism. (4) The study results were written in English.

Only the studies meeting all the above mentioned inclusion criteria would be included in our meta-analysis. In addition, the following articles were excluded in our study. (1)The study did not give a clear pathological diagnosis on NHL. (2) The study did not give a specific number of TP53 mutation in detected cases. (3) The study did not offer the OS or PFS of detected cases, either the K-M curves. The case reports, reviews, meeting abstracts, editorials, or letters to the editor without the original data were not included, and the non-English studies and duplicate articles were excluded.

### Data extraction and assessment of the study

We extracted the following information from the literature: first author, year, country, age of patients, disease subtype, number of total cases and TP53 mutation and wildtype cases, TP53 mutation threshold, study design, and HR (95% CI) of OS or PFS. We evaluated the quality of each study included in our research according to the Newcastle-Ottawa quality assessment scale [12].

### Statistical analysis

We calculated the pooled HR of the studies included in our meta-analysis to explain the correlation between the TP53 mutation status and the prognosis of lymphoma. HRs were extracted for each study directly or indirectly. Five studies provided HRs and corresponding 95% CI of OS or PFS directly. For the studies providing Kaplan-Meier survival curves, data were extracted from the curve through the Enguage Digitizer version 4.1. Thereafter, the HRs and their 95% CI were estimated through the method described in Parmar’s study [13]. The rest of the studies provided the complete patient information on the TP53 mutation status and survival time. Thus, the calculation of HRs was performed with SPSS Version 22.0 software for Windows using the Cox proportional hazards regression model. A calculated HR > 1 suggested a poor prognosis for the group with mutated TP53. The 95% CI of HRs crossing 1 suggested that the correlation between TP53 mutation and prognosis was not statistically significant.

The statistical heterogeneity between the studies included in our document was evaluated through the Chi-square-based Q statistical test based on Peto’s method [14]. A p-value less than 0.10 for the Q-test means that heterogeneity exists among the studies. The Inconsistency index (I^2^) statistic was used to evaluate the level of heterogeneity. A high heterogeneity was related to a large I^2^ statistic. According to the Cochrane systematic reviews, I^2^ ranged from 0%–40%, indicating little or no heterogeneity; 30%–60% indicated rate heterogeneity; 50%–90% indicated substantial heterogeneity; and 75%–100% suggested considerable heterogeneity.

Random-effects or fixed-effects models were selected depending on the heterogeneity among the included studies. The pooled HR was calculated by the fixed-effects model on the condition of mild heterogeneity. Otherwise, the random-effects model was used. Begg’s test and Egger’s test were applied to detect the presence of publication bias. A p-value of less than 0.05 for Egger’s test indicated asymmetry of the funnel plot, whereas a Z-value of less than 1.96 and a p-value of more than 0.05 indicated no publication bias according to Begg’s test. All calculations were performed through STATA version 12.0 software (Stata Corporation, Collage Station, Texas, USA).

## Results

### Literature search and characteristics of studies

**Fig 1:**
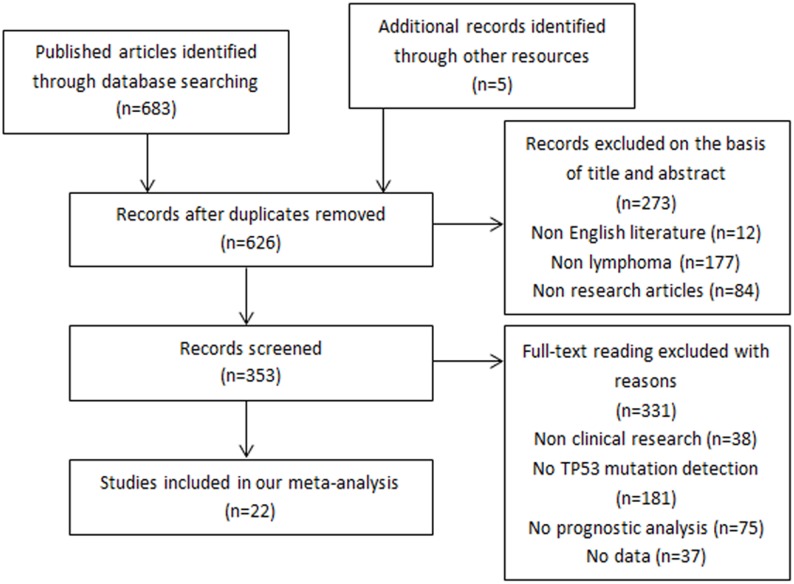
Flow diagram of the relevant studies selection procedure.

We checked 688 possibly related studies using the searching strategy presented in Fig 1. Finally, 22 studies were identified in our meta-analysis [10,11,15–35]. The details of the 22 studies are listed in Table 1. A total of 1,851 patients were enrolled in these studies. Among the 22 studies, 20 provided the HR of the OS, and 5 provided the HR of the PFS.

**Table 1:** Characteristics of studies included in the meta-analysis.

Fisrt Author	Year	Country	Patient (P/N)	Age (year)	Disease subtype	mutation frequency	Therapy regimen	Method	Detected exons	HR (95% CI) of OS	HR (95% CI) of PFS	Study type	Quality score
**Munch-Petersen** [15]	2016	Denmark	86 (24/62)	NA	PCNSL	27.91%	CCT-treated/others	sequencing + DGGE	5–8	1.09 (0.37–3.21)	1.50 (0.75–2.98)	P	8
**Xu-monette** [16]	2012	multiple center	506 (111/395)	NA	DLBCL	21.94%	R-CHOP	sequencing	2–11	1.95 (1.34–2.83)	1.84 (1.28–2.65)	R	9
**Dong** [10]	2011	China	32 (6/26)	59 median	MCL	18.75%	R-CHOP/HyperCVAD/MA/others	PCR-SSCP	5–8	7.39 (1.58–34.65)		R	8
**Rossi** [17]	2011	Italy	85 (31/54)	NA	RS (DLBCL)	36.47%	mainly R-CHOP/CHOP	sequencing	4–8	2.49 (1.41–4.40)		R	9
**Stefancikova** [18]	2010	Czechoslovakia	33 (9/24)	63 median	MCL	27.27%	CT/IT	FASAY/sequencing	5	3.79 (1.51–9.51)		R	8
**O’Shea** [19]	2008	UK	185 (12/163)	NA	FL	6.49%	NA	sequencing	5–8	2.70 (2.30–5.60)	2.70 (2.30–5.60)	R	8
**Young** [20]	2008	multiple center	292 (102/190)	NA	DLBCL	34.93%	CHOP/CHOP-like	sequencing/SSCP/DGGE//DHPLC	5–9	1.70 (1.10–2.80)		P	9
**Norafiza** [21]	2008	Sweden	102 (13/89)	66 median	DLBCL	12.75%	mainly CHOP/VACOP-B	sequencing	4–8		3.56 (1.71–7.42)	R	9
**Young** [22]	2007	Multiple center	113 (24/89)	62.6 median	DLBCL	21.24%	NA	DHPLC	5–8		3.04 (1.69–5.63)	P	8
**Hiraga** [23]	2006	Japan	96 (19/77)	NA	DLBCL	19.79%	Cyclophosphamide + Anthracycline	DHPLC + sequencing	5–9	2.69 (1.27–5.71)		R	9
**Hoshida** [24]	2005	Japan	50 (24/26)	61 median	mainly DLBCL/PTL	48.00%	NA	PCR-SSCP	4–8	2.96 (1.37–6.40)		P	6
**Namiki** [25]	2003	Japan	39 (6/33)	13 median	BL	15.38%	Cyclophosphamide +methotrexate/CHOP	PCR-SSCP	5–9	2.11 (0.69–6.50)		P	7
**Bellido** [26]	2002	Italy	43 (7/36)	55 median	FL/DLCL/BL	16.28%	CHOP/CVP/CHOP + radiotherapy	PCR-SSCP	5–9	5.75 (1.38–23.93)		R	6
**Nakatsuka** [27]	2002	Japan	17 (9/8)	66 median	DLBCL/PTCL	52.90%	chemotherapy/radiotherapy/operation /none	PCR-SSCP	5–8	1.22 (0.39–3.86)		P	7
**Petit** [11]	2000	France	28 (5/23)	69 median	T/NK celllymphoma	17.86%	NA	DGGE + sequencing	5–8	1.00 (0.33–3.00)		R	6
**Stokke** [28]	2000	Norway	70 (8/62)	57 median	B-cell NHL	11.43%	CHOP/CVP/radiotherapy	CDGE + sequencing	5–8	11.91 (4.12–34.43)		P	8
**GRéNBáK** [29]	1999	Denmark	19 (4/15)	65 median	MALT/DLBCL/FCC	21.05%	Chemotherapy/radiotherapy/interferon	sequencing	NA	1.36 (0.31–5.97)		P	7
**Marrogi** [30]	1999	USA	12 (8/4)	48.5 mean	t-CTCL	66.67%	NA	PCR-SSCP +sequencing	5–8	6.01 (0.75–48.42)		P	6
**Baldini** [31–32]	1997	Italy	15 (6/9)	65 median	SMZCL	40%	CEOP/a-2a interferon/none	sequencing	5–8	2.41 (0.67–8.67)		R	6
**Ichikawa** [33]	1997	Japan	102 (22/80)	56 mean	B-cell lymphoma	21.57%	CHOP/CHOP-B/VEPA-M	PCR-SSCP	5–9	3.70 (1.70–8.00)		R	8
**Marks** [34]	1995	USA	18 (2/16)	68.5 median	Sezary syndrome	12.50%	NA	PCR-SSCP +sequencing	2–11	1.23 (0.23–6.52)		R	6
**Preudhomme** [35]	1995	France	21 (6/15)	23 median	BL	28.57%	Chemotherapy	PCR-SSCP +sequencing	5–8	0.88 (0.18–4.37)		R	7

P/N, the number of positive/negative; HR, hazard ratio; NA, not available; R, retrospective; P, prospective; PCNSL, Primary central nervous system lymphoma; DLBCL, diffuse large B-cell lymphoma; MCL, mantle cell lymphoma; BL, burkitt lymphoma; RS, ritcher syndrome; FL, follicular lymphoma; DLCL, diffuse large cell lymphoma; PTCL, peripheral T-cell lymphoma; MALT, mucosa-associated lymphoid tissue-type lymphoma; FCC, follicle centre cell lymphoma; t-CTCL, transformed cutaneous T-cell lymphoma; SMZCL, splenic marginal zone cell lymphoma; R-CHOP, rituximab, cyclophosphamide, doxorubicin, vincristine and prednisone; IT, intensive treatment; CT, classical treatment; PCR-SSCP, polymerase chain reaction-single stranded conformation polymorphism; DGGE, denaturing gradient gel electrophoresis; LLMPP, Lymphoma and Leukemia Molecular Profiling Project; dHPLC, denaturing high performance liquid chromatography.

Among the 22 studies published from 1995–2016, 16 studies were performed in the 21st century, whereas six were conducted in the 20th century. The areas carried out the trails including Europe, North America, and Asia. Three of the studies are multiple-center researches. Four studies were conducted in Japan; three in Italy; two in France, Denmark, and the USA; and one each in China, Czechoslovakia, UK, Sweden, and Norway. The subtypes of lymphoma involved in the studies were various, namely, DLBCL, MCL, Burkitt lymphoma, and others. 1 study investigated the Ritcher syndrome cases, but the RS cases in the study were all classified as DLBCL according to the pathological diagnosis [17]. The detection method of gene mutation in the trails was mainly PCR-SSCP and sequencing. Three studies used DGGE, whereas one study each used FASSAY, CDGE, or DHPLC to detect TP53 mutation. The exons detected mostly ranged from 5 to 8.

The quality score for the selected studies ranged from 6 to 9 through the NOS scoring system, with a median score of 8. A total of eight studies were prospective, whereas 12 were retrospective.

### Pooled HR of OS and PFS

**Fig 2:**
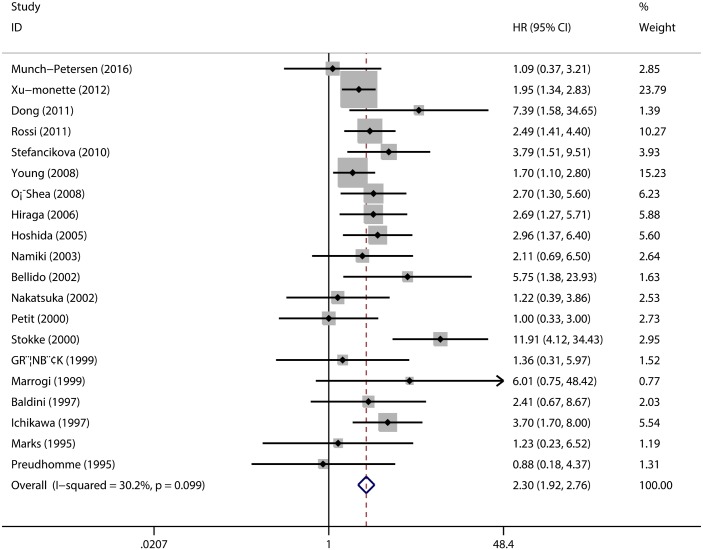
The Hazard Ratio (HR) of TP53 mutation with the Overall Survival (OS). HR > 1 implied worse OS for the TP53 mutated group.

**Fig 3:**
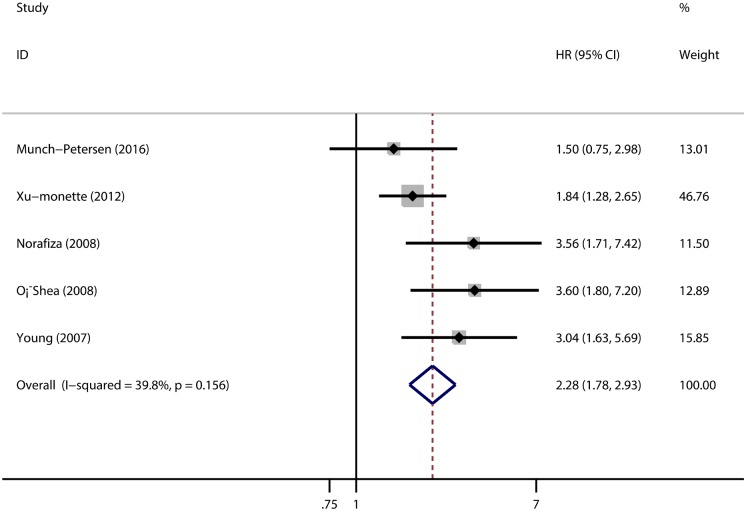
The Hazard Ratio (HR) of TP53 mutation with the Progression Free Survival (PFS). HR > 1 implied worse PFS for the TP53 mutated group.

The pooled HR of the OS from the 20 studies was 2.30 (95% CI: 1.92–2.76, p = 0.001; Fig 2) with heterogeneity (I^2^ 30.2% p = 0.099). The pooled HR of the PFS provided in five articles was 2.28 (95% CI: 1.78–2.93, p = 0.001; Fig 3) with heterogeneity (I^2^ 39.8% p = 0.156). Both results indicate that the mutations of TP53 predict a poor prognosis on NHL.

**Fig 4:**
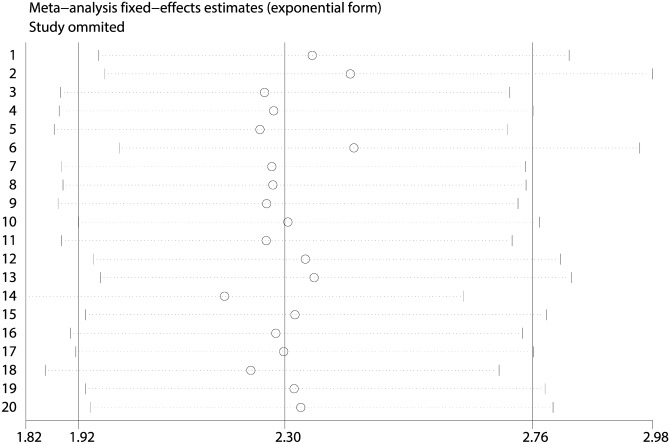
The sensitivity analysis of the Overall Survival (OS).

Sensitivity analysis of OS was performed (Fig 4), and the outcome demonstrated that the removal of any of the studies would not have an important effect on the combined HR. Thus, a negative correlation between mutated TP53 and the prognosis of NHL existed after any study was excluded from our meta-analysis.

### Analysis on the pooled HR of OS in the subtypes of NHL

**Fig 5:**
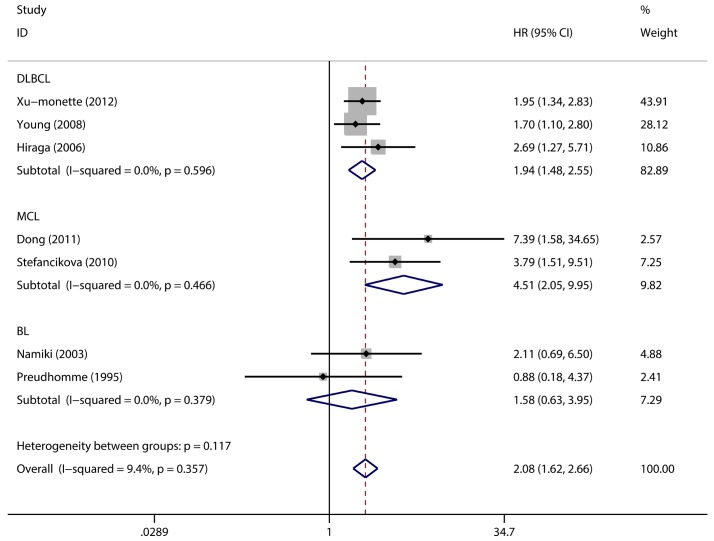
The Hazard Ratio (HR) of TP53 mutation with the Overall Survival (OS) of different subtypes. HR > 1 implied worse OS for the TP53 mutated group.

An analysis on the subtypes of NHL was performed. The pooled HR of the OS provided in three articles concerned with DLBCL was 1.94 (95% CI: 1.48–2.55, p = 0.001; Fig 5). The pooled HRs for MCL and BL were 4.51 (95% CI: 2.05–9.95, p = 0.001) and 1.58 (95% CI: 0.63–3.95, p = 0.329), respectively (Fig 5).

### Subgroup analysis for OS

We performed subgroup analysis of OS on the publication year, areas, quality score, study design and number of total patients (Table 2). The findings suggest that the negative association between TP53 mutation and the survival time of NHL existed in all subgroups that we listed.

**Table 2:** Subgroup analysis of the pooled HRs with Overall Survival (OS).

					Heterogeneity	
stratified analysis	Number of studies	Number of patients	Pooled HR (95% CI)	P value	I^2^ (%)	P value
**Published era**						
**Published in 21st**	14	1562	2.282 (1.878–2.773)	0.001	42.30%	0.047
**Published in 20th**	6	187	2.428 (1.445–4.078)	0.001	0.00%	0.461
**Study location**						
**Europe and America**	12	615	2.733 (1.940–3.850)	0.001	42.20%	0.061
**Asia**	6	336	2.815 (1.934–4.097)	0.001	0.00%	0.506
**Number of patients**						
**≥ 50**	9	1472	2.543 (1.887–3.426)	0.001	49.00%	0.047
**< 50**	11	277	2.197 (1.485–3.251)	0.001	12.90%	0.321
**Quality score**						
**≥ 8**	10	1487	2.395 (1.948–2.944)	0.001	51.30%	0.030
**< 8**	10	262	1.991 (1.349–2.938)	0.001	0.00%	0.525
**Study design**						
**P**	8	585	2.146 (1.570–2.932)	0.001	54.50%	0.031
**R**	12	1164	2.384 (1.904–2.984)	0.001	4.90%	0.397

### Publication bias

**Fig 6:**
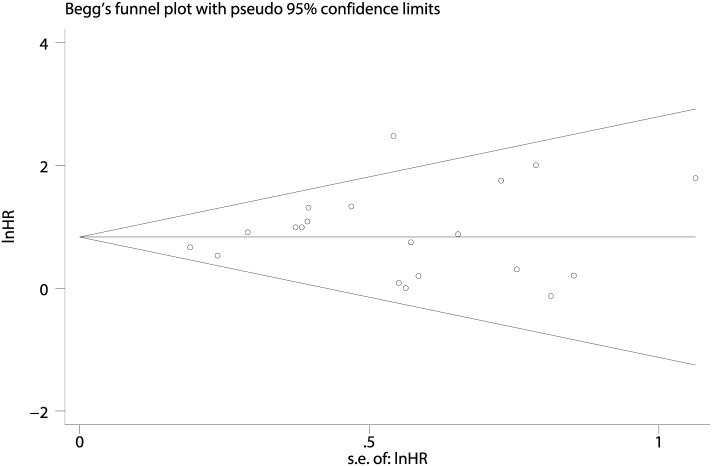
The funnel plot of the Overall Survival (OS) of NHL.

Egger’s test indicated that the funnel plot was symmetrical (p = 0.422), and the p-value of Begg’s test was greater than 0.5 (p = 0.604, Z = 0.52). Both outcomes suggested no publication bias for the studies included in our meta-analysis. The funnel plot of Begg’s test was presented (Fig 6).

## Discussion

According to our statistical data, 1,851 patients were examined, and the mutation frequency among the studies ranged from 11.43% to 66.67%, which was approximately distributed across the disease subtypes and influenced by the statistical significance of patients. The combined HR of OS in the 22 studies included in our meta-analysis was 2.30 (95% CI: 1.92–2.76, p = 0.001) with heterogeneity (I^2^ 30.2% p = 0.099). The Q test and I^2^ test revealed low heterogeneity of the study. The results indicated that TP53 mutation negatively affected the survival time of patients with NHL.

The aberration of chromosome 17 in patients with lymphomas is related to drug resistance, therapeutic failure, and high tumor-related mortality rate [36]. Thus, the status of TP53 localized in chromosome 17 may influence the progression and survival of lymphomas. TP53 mutations have been detected in various human organs and in more than 50% of human cancers [37]. The most important domain of TP53 is the DNA-binding domain (DBD), and most TP53 mutations are missense mutations occurring in the DBD encoded by exons 4 to 8 of TP53 [22], [37]. In our meta-analysis, the trials for the detection of TP53 mutation mostly involved these exons. The high frequency of these mutations can disrupt the TP53 structure and then deactivate the TP53 pathway [38]; as a consequence, disruptions in cell cycle, DNA repair, apoptosis signaling, and apoptosis and autophagy in the cytoplasm occur, and these processes are related to tumor development and progression [9]. The dominantly negative effect exhibited in mutant TP53 can cause adverse therapeutic outcomes and early cancer recurrence [39]. Several studies included in our research also confirmed that TP53 mutations in the DBD are significantly correlated with poor OS in patients with NHLs [15,16,22]. In 2014, the American Association for Cancer Research (AACR) recommended two categories of precision medicine: Umbrella Trails and Basket Trails. Basket trails aim to investigate the therapeutic value of the same target genes in different types of cancers or human organs. Our results indicated that TP53 could be a potential target gene for precise individual therapeutic regimen.

Disease subtype analysis implied that the prognostic value of TP53 mutation was distinguished in the different subtypes of NHL. The pooled HR of DLBCL was 1.94 (95% CI: 1.48–2.55, p = 0.001), and a total of 894 patients were included in three studies. DLBCL was the most common type of NHL that is highly heterogeneous. The pooled HR confirmed the prognostic significance of TP53 mutation in DLBCL and indicated the clinical value of this phenomenon. By comparison, the pooled HR of MCL was 4.51 (95% CI: 2.05–9.95, p = 0.001). Several studies have confirmed that TP53 mutation is closely involved in advanced MCL, and the frequency of TP53 mutation reaches 27.3%, as revealed by a FASAY method [18]. Our result showed that TP53 mutation was strongly correlated with poor MCL prognosis, although the total cases were small in the two investigated articles. This result could be influenced by different sensitivities of the testing methods and the small sample size. Thus, the gap between the pooled HRs of DLBCL and MCL should be further investigated.

The combined HR showed the prognostic significance of TP53 mutation in NHL, but some inconsistent results were found in our investigation. The correlation between TP53 mutation and prognosis was not statistically significant in eight studies because of the 95% CI of HRs crossing 1. A study implied that TP53 mutation does not exhibit an intergroup difference in T/NK cell lymphomas [11]. The combined HR of Burkitt lymphoma in subtype analysis was also not significant probably because of the insufficient number of samples and unified therapies. Different treatment regimens could significantly affect the prognosis of NHL, which showed remarkable heterogeneity among different subtypes. However, the studies included in our meta-analysis did not strictly treat patients with the unified therapy strategy, and the lymphoma subgroups were analyzed on the basis of few articles because of the clinical characteristics of the included studies. Thus, the investigation based on separate subgroup analyses and R-CHOP-treated NHLs should be improved.

In conclusion, this meta-analysis supports the prognostic value of TP53 mutation despite the limitations of our study. TP53 mutation is considered a prognostic indicator for NHL. Further studies on the prognostic value of single NHL subtypes and therapeutic effects should be conducted to verify our findings.

## Supporting information

S1 PRISMA Checklist PRISMA 2009 checklist. (DOC)
